# Chronic kidney disease in hypertensive patients: the urgent need for targeted interventions in Arab countries: a systematic review

**DOI:** 10.3389/fneph.2026.1735217

**Published:** 2026-02-09

**Authors:** Fakhria Al Rashdi, Celine Tabche, Zeenah Atwan, Hamed Al-Qanubi, Samiya Al Khaldi, Nasrin Al-Zadjali, Salman Rawaf

**Affiliations:** 1 Ministry of Health, Muscat, Oman; 2 WHO Collaborating Centre for Public Health Education and Training, School of Public Health, Imperial College London, London, United Kingdom; 3 Central Laboratory, College of Medicine, University of Basrah, Basrah, Iraq

**Keywords:** Arab countries, chronic kidney disease, hypertension, prevention, primary care

## Abstract

**Background:**

Chronic kidney disease (CKD) is expected to be the 5th leading cause of years of life lost by 2040. Recently, it emerged as a significant cause of mortality and morbidity, with a high prevalence in Arab countries.

**Objective:**

Assess CKD among hypertensive (HTN) people in Arab Countries through evaluation of the existing literature on CKD prevalence, risk factors, screening programmes and prevention.

**Study design:**

A systematic review till April 2024 following PRISMA guidelines. The search strategy was registered in PROSPERO under the identification code CRD42024486068.

**Methods:**

Databases searched were Medline, Embase, Scopus, PubMed, Cochrane Library. Screening was done using Covidence by three independent reviewers.

**Results:**

Out of 63 studies screened, 11 were selected for extraction. The prevalence of CKD was higher among elderly, HTN and diabetic patients, with 38.8% having unrecognised CKD. Nearly 39% of the 400 participants in one study had undiagnosed stages 3–5 CKD. Two studies showed that 55.8% and 75% of identified CKD patients had HTN. Physicians reported suboptimal screening rates, with about 77% relying on the estimated glomerular filtration rate as a diagnostic tool. Risk factors for CKD include old age, HTN, dyslipidaemia, family history of CKD, and obesity. Among physicians, 85% recommended a target blood pressure of ≤130/80, 80% advised smoking cessation, 66% prescribed anti-lipids, and 67% recommended weight loss. All studies support the fact that HTN is a risk factor for CKD.

**Conclusion:**

CKD is an escalating problem in Arab countries, with hypertension as a major risk factor. Many patients remain undiagnosed. A region-specific CKD screening and HTN control programme is urgently needed. The findings are essential for policymakers in strengthening primary care for systematic screening of HTN and CKD.

**Systematic review registration:**

https://www.crd.york.ac.uk/prospero/, identifier CRD42024486068.

## Introduction

Chronic kidney disease (CKD) is one of the most significant causes of death and suffering globally in the 21st century (1). According to the Global Burden of Disease (GBD) 2021 study, CKD affects an estimated 697.5 million cases worldwide and has a prevalence rate of approximately 9.1% (2). It is predicted to be the fifth leading cause of the years of life lost by 2040 (3). Economically, the cost of dialysis and transplantation accounts for up to 3% of the annual healthcare budget in high-income countries (3).

CKD is diagnosed when the estimated glomerular filtration rate (eGFR) is below 60 mL/min/1.73 m², albuminuria (at least 30 mg/24h), or the persistent presence of markers of kidney damage for over three months, according to the National Institute for Health and Care Excellence (NICE) and the Kidney Disease Improving Global Outcome (KDIGO) (4, 5). Once CKD is diagnosed, it is classified into five stages based on eGFR (G1–G5) and urinary albumin levels (A1-A3). Individuals with normal or slightly reduced eGFR (G1 or G2) and minimal albuminuria (A1) are at low risk, while those with eGFR below 60 ml/min/1.73 m² and moderate albuminuria face a higher risk (4). CKD is more commonly observed among diabetics, hypertension (HTN) patients, older adults, women, and racial minority groups (1). CKD could be a cause or consequence of HTN (6). HTN is a major global risk factor for cardiovascular disease (CVD) and is strongly linked to CKD. Among hypertensive adults in the United States, CKD prevalence was 35.8% (2011–2014), compared to 14.4% in prehypertensive individuals and 10.2% in those without HTN (7). A meta-analysis of 75 global studies further validated this strong association (7, 8). Disability-adjusted life years (DALYs) associated with CKD caused by HTN increased from 286 in 2019 to 288.7 in 2021 (2).

Growing awareness of early-stage CKD emphasises the crucial role of primary care clinicians in identifying the condition, assessing risk, and collaborating with patients to optimise management and reduce complications (4). CKD can be prevented through effective preventive strategies, categorised into three levels: primary, secondary, and tertiary (3). Primary prevention by reducing disease occurrence through early screening and CKD detection in individuals with chronic conditions (3). Secondary prevention emphasises early diagnosis and treatment, health education and clinical interventions to slow the disease progression. Additionally, controlling blood pressure (BP) is crucial in preventing ongoing kidney damage (3). Tertiary prevention involves managing the disease to slow progression and prevent severe complications (3). A structured, patient-centred approach, including lifestyle modifications, effective BP control, and the careful selection of antihypertensive medications, can help slow CKD progression and reduce CVD risk (6). Whilst many countries have integrated CKD screening into their non-communicable disease (NCD) programmes, the strength and effectiveness of these programmes vary. Examples of these programmes are the NCD initiatives implemented by the UAE at several national levels, including healthy school canteen guidelines, adult screening programmes since 2014, and the incorporation of NCDs into climate and environmental strategies (9). Furthermore, Oman launched a national screening programme for citizens aged 40 and above, which was piloted in 2006 and expanded nationwide in 2007, thereby enhancing early detection of NCDs (9).

In the Arab countries, CKD-related deaths and DALYs have steadily increased since 1990, highlighting challenges in early detection and management (10). Data from the GBD 2021 indicated a 36.7% increase in age-standardised deaths due to CKD in the Arab countries between 1990 and 2021, and DALYs due to CKD in all ages increased by 45% (10). The Arab region comprises 22 developing countries, with a total population of approximately 492 million, according to the World Bank (11). These nations include Algeria, Bahrain, Comoros, Djibouti, Egypt, Iraq, Jordan, Kuwait, Lebanon, Libya, Mauritania, Morocco, Oman, Palestine, Qatar, Saudi Arabia, Somalia, Sudan, Syria, Tunisia, the United Arab Emirates, and Yemen. Data from GBD 2021 found that CKD incidence in Arab countries was 403 per 100,000 people, nearly double the global rate of 233 per 100,000 (10). While these countries have socioeconomic differences, they share a common language, culture, and religion. Many Arab countries with high CKD prevalence are inadequately prepared to address the consequences (1).

Although the CKD burden has been described at regional and global levels, evidence specifically quantifying CKD prevalence and related screening/management gaps among hypertensive adults in Arab countries remains fragmented across settings and study designs (5, 10). The study aims to evaluate and review the existing literature on the prevalence of CKD among patients with HTN, including risk factors, screening programmes, prevention strategies and policy implications in Arab countries.

## Methods

### Research question

Our research question was: “What is the prevalence, risk factors, and gaps in screening and management of CKD among hypertensive patients in Arab countries, and how can targeted interventions address these challenges?” To address this, we conducted a systematic review of the literature on CKD prevalence, risk factors, screening, and management among hypertensive adults in Arab countries. The search strategy was registered in PROSPERO under the identification code CRD42024486068.

### Search strategy

Searches were conducted in Embase, Scopus, PubMed, and Cochrane Library. No date restrictions were applied; databases were searched from inception to April 16, 2024. MeSH terms and free text were used (**Supplementary file 1**). Due to heterogeneity in population, intervention, and comparator, no meta-analysis was performed. Where available, reported effect measures (odds ratios or incidence rate ratios) were extracted; however, the data were not sufficiently standardised to support additional consistent calculation of risk ratios across studies or pooled estimates.

### Study selection

**Figure 1:**
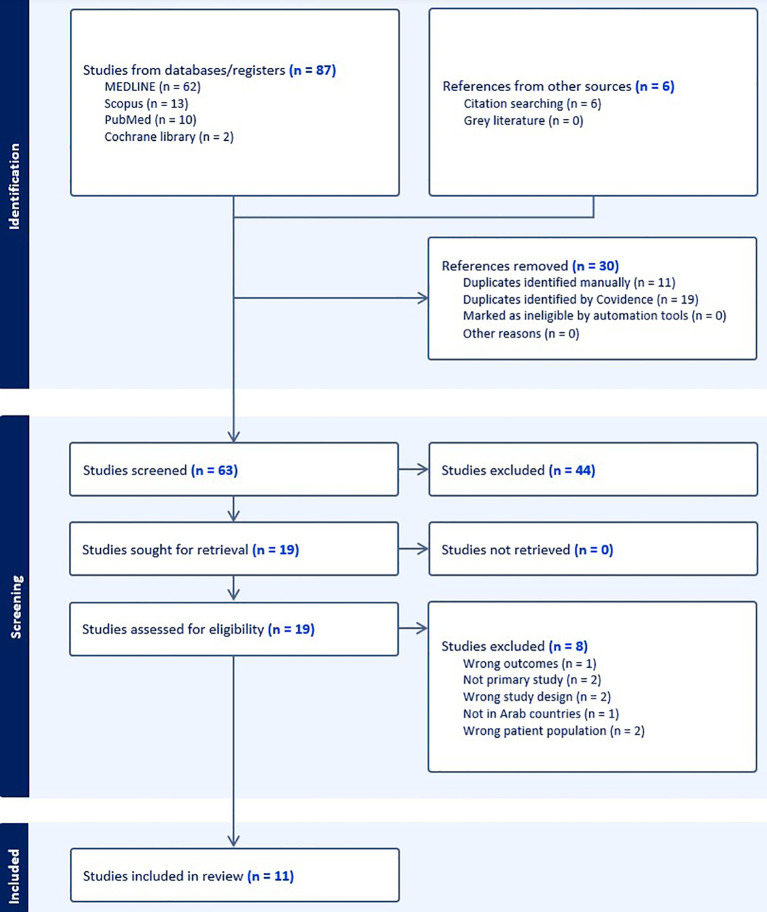
PRISMA flow diagram.

The included studies were case-control studies, randomised controlled trials (RCTs), non-RCTs, and cohort studies. These studies focused on the management of HTN and its impact on the prevalence and progression of CKD. We included full-text English/Arabic original studies (case–control, cohort, RCT/non-RCT) in adults (≥18 years) in Arab countries that examined hypertension interventions and/or compared CKD prevalence/prevention/treatment among hypertensive populations and reported CKD-related outcomes. We excluded paediatric studies, non-original publications (systematic reviews, editorials, commentaries, conference abstracts), studies without accessible full text, non-English/Arabic papers, and studies where CKD outcomes were not linked to hypertension. Detailed inclusion and exclusion criteria are listed in **Table 1**. This study adhered to the guidelines of the Preferred Reporting Items for Systematic Reviews and Meta-Analyses (PRISMA) statement (12), as outlined in **Figure 1**. Title/abstract screening and full-text eligibility assessment were conducted independently by two reviewers. Discrepancies were resolved through discussion, and if unresolved, were adjudicated by an independent third reviewer.

**Table 1:** Inclusion and exclusion criteria used for screening the articles.

PICOS	Inclusion criteria	Exclusion criteria
Population	Population aged 18 and above in Arab countries.	Population age below <18 in Arab countries.
Intervention	Hypertension intervention programs with outcomes related to kidney disease.	Hypertension intervention programs are not related to kidney disease.
Comparator	Studies that compare CKD prevalence, treatment and prevention among HTN patients	Studies that compare CKD prevalence, treatment and prevention among HTN patients
Outcome	CKD prevalence, risk factors and role of hypertension treatment to prevent CKD. Evaluating the barriers to controlling BP and kidney disease progression in Arab countries.	Studies about CKD are not related to hypertension.
Language	Articles with full text and published in English and Arabic.	Articles with full text are not found or published in English or Arabic.
Type of studies	Case-control studies, RCT or non-RCT, Cohort studies)	Systematic reviews, editorials, commentaries, and conference abstracts.

### Data extraction

After reviewing the full texts of eligible studies, data extraction was performed by two independent reviewers (**Table 2**). The extraction included the following themes: Prevalence and Incidence of CKD, screening methods, risk factors, management of CKD and HTN. We also extracted how each study defined and ascertained hypertension (documented diagnosis, antihypertensive treatment, or measured BP thresholds) and CKD (eGFR thresholds and whether albuminuria/proteinuria testing was included). Variability in these definitions was recorded and considered when interpreting between-study differences.

**Table 2:** Overview of the characteristics of the included studies and a summary of their results.

Study	References (author and year of publication)	Country	Type of study and duration	Setting (Government, Private, School, others)	Targeted group and age	Outcome measure	CKD management among HTN
1	AlShamsi 2016	United Arab Emirates.	Cross-sectional study 26 September 2013 to 5 March 2014.	Primary and specialised healthcare facilities of the public and private sectors	Non-nephrologist physicians 135 non-nephrologists (PHC+SHC)	The physicians’ approach to identifying and managing patients with CKD.	Screening for CKD: HTN: 94% for CKD Using eGFR: 77% using serum creatinine: 19.3% using ACR:59% both eGFR +ACR: 49.6% referral: 55%: eGFR <60 or CR >150 umol/L 59%: if there is significant proteinuria 25%: if there is association with hematuria Management: 96% CKD is at risk for CVD but 66%give antilipid trx 67%: weight reduction 80%: smoking cessation 85%: a blood pressure control ≤130/80 as an optimal target, 13%: use a blood pressure target of >130/80 and 1.5% have no specific blood pressure target. 76%: initiate ACE inhibitors or angiotensin receptor blockers (n=103), but in practice 61% of those who offer ACE inhibitors estimated that at least half of their patients were on these medications. PHC doctor is better than SCH in use of EGFR in DX of CKD. PHC better in initiation of ACR than SHC
2	Hassanien 2014	Saudi Arabia	Retrospective observational study first date of initiating hemodialysis to the end of 2011.	hospitals	208 patients with end-stage renal disease on regular hemodialysis in 2011.	- The primary reasons for hospital admissions and - Risk factors lead to hospital admissions and increase the length of stay in hospital.	Dominant cause of renal disease in the study sample: - Hypertensive nephropathy (58.8%), - Diabetic nephropathy (23.9%) - Glomerulonephritis (4.1%). prevalence of co-morbid conditions was high, particularly: Diabetes (24.8%) and HTN (75.2%), hepatitis B (8.5%) and hepatitis C (47.5%) The length of stay in hospital: between 1 and 80 days per hospital admission with a median of three days per admission (25%, 75% percentiles: 2, 6). The most frequent reasons for hospitalisation: - Genitourinary system (40.1%) - Diseases of the circulatory system (22.6%) -Vascular access (19.5%). -Overall infectious diseases (9%) Patients with ESRD due to glomerulonephritis or diabetic nephropathy had 2.28-fold and 1.84-fold higher admission rates, respectively, compared to those with hypertensive nephropathy, after adjusting for age, sex, and access type.
3	Nassr 2019	Iraq	Prospective Cross-sectional Study. April 2018 and August 2018,	Hospital	224 adult patients with HTN attending primary care clinics	- To evaluate the extent and determinants of patient BP control and examine antihypertensive prescribing patterns.	- 38.7% of patients had controlled BP. - No difference in BP control was found based on education, employment, smoking, non-diabetic comorbidities, or treatment regimen. - Younger males (<60 years) were twice as likely to have uncontrolled BP. - Diabetics were more than three times as likely to have uncontrolled BP. - ACEIs or ARBs were the most prescribed antihypertensives (74.7%).
4	Nasser 2010	Bahrain 1st of April till 30th of June 2009	A retrospective clinical study	Primary care	773 pt, Hypertensive patients	level of control and pattern of prescribing for people with hypertension	- Overall BP control rate: 35.8% (205/573). - Lower control in diabetic patients: Dropped to 26.6% (72/271) in those with co-morbid diabetes. - Poor BP control in older adults: Low rates in patients over 60, regardless of the status of diabetes. - Common coexisting conditions: 47.5% (272/573) had dyslipidemia, and 10 patients had CKD. - Gender differences in BP control: Better in females (41.1%; 145/353) than males (27.3%; 60/220). - Persistent low control in older adults: Both sexes had poor BP control over 60 years.
5	Jairoun 2024	UAE	prospective point-of-care interventional study from January 1st to November 30th, 2023	community pharmacies	Individuals aged 18 or older are at risk for CKD if they meet at least one of the following KDIGO criteria: - Diabetes (type 1 or 2) - HTN - Family history of kidney disease	Assessing CKD prevalence in high-risk patients and evaluating the impact of a community pharmacist-led point-of-care screening program	- Mean eGFR: 76.3 ± 27 mL/min/1.73 m². - Comorbidities: Vascular disease (45%; n=180), hypertension (86.5%; n=346), diabetes mellitus (32.3%; n=129). - Undiagnosed CKD: 38.8% (n=155) had unrecognised CKD. - CKD stages 3–5 prevalence linked to: - Older age (OR 1.099, 95% CI 1.075–1.123, p<0.001). - Hypertension history (OR 1.66, 95% CI 1.015–2.73, p=0.043). - Diabetes history (OR 2.86, 95% CI 1.85–4.41, p=0.001)
6	S Al-Shamsi2018	UAE	retrospective from 2008, -30 June 2017(8.6 yrs)	primary care &Tawam Hospital outpatient clinics in Al Ain,	491 UAE nationals, age ≥ 20 years with CVD or a high CVD risk	-Incidence and causes of chronic kidney disease stages 3–5	Total incidence: 11.4% in 8 year Incidence rate: 164.8 cases per 10,000 person-years. Risk Factors in Males: Older age. History of Coronary Heart Disease (CHD). Diabetes Mellitus (DM). Vascular disease. Hypertension (HTN). Dyslipidemia. Risk Factors in Females: Older age. Diabetes Mellitus (DM). Hypertension (HTN). Obesity. Adjusted Hazard Ratios (HR) for Risk Factors: The history of CHD increased risk by 2.5 times. Diabetes increased the risk nearly fourfold. History of smoking increased risk by 2.4 times.
7	Ahmed Abdulrahman Aldhahi1*	Saudi Arabia	Prospective cross-sectional survey of	Hospital	712 patients with hypertensive, age >16	prevalence and staging of CKD	Blood Pressure: Median BP 150/90 mmHg, HTN duration 4 years (range 0.1–50). Median HTN drugs: 2 (range 0–5). Hypertensive Medications: ACE inhibitors/ARBs (25.1%), CCBs (83.1%), Beta-blockers (35.8%), Diuretics (55.3%). CKD Prevalence: Overall, 46.9%. Stages 1–2 (eGFR ≥ 60 with proteinuria): 19.1%. Stages 3–5 (eGFR < 60): 27.8%. Proteinuria: 28.9% (95% CI: 25.6–32.4). Diabetes & CKD: No significant difference (p = 0.133). Other Findings: Advanced CKD is more common in young patients (p = 0.0001). Proteinuria higher in stages 1, 2, and 4 vs. stages 3 and 5. No significant correlation between CKD and age (p = 0.901). Few cardiovascular events, not linked to CKD severity
8	Hussain Gadelkarim Ahmed1	Saudi Arabia	cross sectional survey March 2012 to October 2013	13 towns around Hail city	2800 full respondents, 217 have CKD Saudi civilian +mean age 45 yrs	screen for CKD and HTN in 13 towns around Hail city	CKD Prevalence:217 participants (7.8%) with CKD. Stages identified: Stage III: 90.3% (196/217, GFR=59–30 mL/min/1.73m²). Stage II: 5% (11/217, GFR=29-16 mL/min/1.73m²). Stage I: 4.7% (10/217, GFR=15 ≤ mL/min/1.73m²). Hypertension Prevalence: Total hypertension: 939/2800 (33.4%). Males: 477/939 (50.9%). Females: 462/939 (49.1%). CKD and Hypertension: Of the 217 patients with CKD, 121 (55.8%) had hypertension. Hypertension as a risk factor for CKD development was statistically significant (P value < 0.0001).9
9	Hala H. Sa’adeh1	Palestine	cross sectional study January 2016 until February 2017.	3 primary healthcare centers in Nablus, Palestine	374 hypertensive patients from primary healthcare centers in Nablus, Palestine, aged ≥18 years, with HTN >6 months, fluent in Levantine Arabic.	Knowledge, attitudes and practices of hypertensive patients towards prevention and early detection of chronic kidney disease	higher knowledge scores: Age < 65 years Normal BMI High education level High number of medications higher attitude scores: Age < 65 years Employed status Married status High income High education level higher practice scores: Male gender Normal BMI High income High education level High number of chronic diseases High number of medications
10	Amani A. Khalil	Jordan	a cross-sectional, September 2013 to March 2014.	12 hospital outpatient clinics	540 pt high risk for CKD Age >18, understand Arabic, at risk for CKD (DM/HTN/family History of renal disease)	prevalence of CKD at high risk pt + association of CKD with demographic and clinical factors	Majority were females: 64%. Mean age ( ± SD): 55.0 ± 12.5 years. eGFR Statistics: Mean eGFR ( ± SD): 116.0 ± 47.5. eGFR distribution: Mild reduction: 23.5%. Moderate reduction: 5.4%. Severe reduction: 0.7%. A very severe reduction: 0.7%. Risk factors for CKD Development: Ageing/Male gender/Unemployment/Packs/years of smoking Comorbidities: (HTN), diabetes mellitus (DM), cardiovascular disease Low high-density lipoprotein (HDL) No statistically significant difference in eGFR across the four groups (pt on ACEI/ARBs& NSAIDs) -Approximately 31% of the individuals had an unrecognised CKD.
11	Ali Manal Kamil1	Iraq	cross-sectional study, October 2019-February 2020	dialysis center and hospitals	(273) cases of chronic kidney disease	Prevalence of HTN, CKD and find the relationship between them	CKD Prevalence in Al Basra province: 6.8% Hypertension: 75.72% of patients had HTN before CKD. (43.58%) males had before CKD (1.4%) had after CKD. 25.64 females had before CKD, and 5.1% had after. Significant positive association between hypertension and CKD.

### Assessment of studies’ quality

The Newcastle-Ottawa Scale (NOS) critical appraisal checklist was used to assess the quality of the included studies and ensure their methodological accuracy (**Supplementary file 2**). All the articles successfully passed the quality assessment. This allowed us to indicate a low risk of bias across the studies, supporting the reliability and robustness of the evidence presented in this review.

## Results

A total of 87 studies were initially registered. Six studies were included from manual searching. Among these, 30 duplicates were identified and removed. The remaining 63 studies underwent a title and abstract screening. A full-text review was conducted for 19 studies. An independent third reviewer resolved any disagreements. Eight studies were later excluded for various reasons outlined in the PRISMA. Ultimately, 11 studies were included in the final review. Most were cross-sectional surveys or audits conducted in primary care, outpatient clinics, or community settings, while two studies examined dialysis or end-stage renal disease populations.

### Prevalence and incidence of CKD

Six studies (13–19) pointed to the prevalence and incidence of CKD. A significant increase in the trend of CKD stages 3 to 5 was reported by Jairoun et al. in 2024, with the increased age, hypertension and diabetes mellitus (DM). In addition, he noted that 38.8% had unrecognised CKD (13). Furthermore, a similar finding was reported in a study conducted in Jordan, where 31% of the individuals had unrecognised CKD (16).

The prevalence of HTN in two KSA studies was almost 47%, of which 27% were at stages 3–5. Furthermore, it was shown that CKD prevalence reached 7.8%, and around 55.8% were hypertensive (p-value = 0.0001) (14, 15). Another study in Iraq showed a 6.8% prevalence of CKD in the Basra province (17). In Al-Shamsi’s study, on the UAE nationals (n=491), the total incidence for stages 3–5 was 11.4% over the years till 2017, the incidence rate was 164.8 per 10,000 person-years (18).

Differences in study design and sampling frames likely explain part of this variation. Studies restricted to hypertensive clinical populations tended to report higher CKD prevalence and a greater proportion of advanced stages than community surveys, which included broader populations and then assessed overlap between CKD and hypertension (14, 15). Similarly, studies based in dialysis centres or among haemodialysis patients are not comparable to community or primary care estimates, but they highlight the heavy comorbidity burden and hypertension’s prominence among advanced kidney disease populations (17, 20). These contrasts underscore the importance of interpreting prevalence in the context of the underlying setting (primary care, community, outpatient clinics, dialysis centres) and the population profile (hypertension-only versus high-risk or established CKD).

### Screening methods

Three studies pointed to the validity and effectiveness of screening methods in identifying CKD patients with management guidance and referral (13, 16, 18). The study by Al Shamsi, conducted in the UAE, showed that almost all non-nephrologist physicians will test patients for CKD when they have hypertension and DM (18). However, elderly and CVD patients are tested less for CKD. A suboptimal screening rate was reported by physicians, with about 77% relying on eGFR as a diagnostic tool. In the Jairoun et al., UAE study, the eGFR was provided by the community pharmacist within 10 to 15 minutes on-site using a PICCOLO device. The study showed that almost 39% of the 400 participants have undiagnosed stages 3–5 CKD (13). In Jordan, Amani et al. found that approximately 31% of individuals had unrecognised CKD, a situation compounded by their further observation that 92% of participants had not undergone an albumin test, contributing to the underdiagnosis of CKD (16).

### Risk factors

HTN has been recognised as a risk factor for the progression of CKD in several studies (13, 14, 16, 19–21). Similarly, DM has consistently been identified as a significant contributor to CKD development, alongside other predictors such as CVD and age (13, 16, 19, 20).

Moreover, studies by Al-Shamsi et al. and Amani et al. highlighted additional risk factors, including smoking, being male, unemployment, and low HDL levels (16, 18). Additionally, Al-Shamsi’s study found that age, coronary heart disease, DM, and smoking were significant predictors of CKD, with HTN identified as a risk factor at a p-value of 0.02 (19). Jairoun’s research underscored the importance of age, HTN, CVD, and DM as significant predictors of CKD, with age and DM being particularly associated with a higher incidence of the disease (13).

Both diabetes and HTN were shown to be risk factors for CKD, with incidence rate ratios (IRRs) of 2.55 and 1.84, respectively, which also correlated with increased hospital admissions (20). In Iraq, a 2019 study by Nasser et al. revealed that less than 40% of the population achieved controlled BP, highlighting it as a risk factor for both renal and CVD (22). Finally, Kamil et al. reported that 75% of CKD patients were hypertensive (17).

### Management of CKD and HTN

The study by Al Shamsi et al. (2016) reported that an optimal BP target of ≤130/80 mmHg was recommended by 85% of physicians. Additionally, 80% of them advocated smoking cessation, while 66% and 67% recommended lipid-lowering therapy and weight loss, respectively. Notably, around 66% of physicians prescribed antilipid treatments, though approximately one-third did not favour the use of angiotensin inhibitors (18). Two studies from Bahrain and Saudi Arabia reported BP control rates among hypertensive patients of 36% and 38% (14, 23), with gender differences observed, BP control was better in females (41.1%) compared to males (27.3%) (23).

In another study (13), BP management strategies involved the use of angiotensin II receptor blockers in 74.7% of cases and angiotensin II converting enzyme inhibitors in 29.3% of cases, respectively. Beta blockers, calcium channel blockers, and diuretics were utilised at rates of 28% and 23%, respectively (13).

Collectively, these studies highlight hypertension as a key risk factor for CKD. A study conducted by Sa’adeh et al. (2018) in Nablus, Palestine, further indicated that knowledge, male gender, and a body mass index (BMI) below 25 are protective factors for CKD prevention in hypertensive individuals (21).

## Discussion

This review examined the prevalence, risk factors, screening practices, and management of CKD among individuals with HTN in Arab countries. The findings indicate a concerning burden of CKD among HTN populations, often complicated by late-stage diagnosis, suboptimal screening practices, and disparities in care delivery. Across the included studies, the prevalence of CKD was consistently higher among individuals with HTN, DM, and advanced age (13, 20, 22). For example, studies from Saudi Arabia reported CKD prevalence of approximately 47% among hypertensive patients, with 27% identified in stages 3–5 (14, 15). These findings are in line with those of Alshehri et al. (2025), who also reported stage 3 CKD as the most common form, albeit with a lower overall prevalence of 4.76% (24). In the UAE, Al-Shamsi et al. (2018) found a cumulative incidence of CKD stages 3–5 of 11.4%, translating to 164.8 cases per 10,000 person-years (19). More recently, Jairoun et al. (2024) highlighted a rise in stage 3–5 CKD cases in the UAE, driven by ageing, hypertension, and diabetes, with nearly 39% of cases previously undiagnosed (13).

The broader global context supports these findings. The GBD 2021 study reported an increase in CKD prevalence in Arab countries from 9.06% in 2000 to 9.24% in 2021, exceeding the global average of 8.32% (10). Notably, Arab men and women with hypertension are significantly more likely than their white counterparts to progress to end-stage renal disease (25). Gender disparities in disease progression are well documented, with men exhibiting higher CKD mortality and DALYs despite a higher prevalence in women (26, 27). Over the past fifty years, Arab countries have undergone significant shifts in health and nutritional conditions (28). Diet-related chronic illnesses, including obesity, diabetes, HTN, cardiovascular diseases, and certain cancers, have become widespread health concerns (29).

Screening practices remain inconsistent, despite clear recognition among healthcare professionals of the need to screen hypertensive and diabetic patients for CKD. Al-Shamsi et al. (2016) found that while non-nephrologist physicians in the UAE generally screen these high-risk populations, rates were lower for elderly patients and those with cardiovascular disease (18). Importantly, screening strategies often rely solely on eGFR, with limited use of albuminuria testing (16). Similarly, it was found that 92% of participants had not undergone an albumin test, a diagnostic gap that risks underestimating CKD prevalence (16). This issue is particularly critical given that KDIGO (2024) guidelines emphasise the combined use of eGFR and albuminuria for accurate diagnosis and risk stratification (30).

Emerging models of community-based screening offer promising alternatives. For instance, Jairoun et al. (2024) demonstrated that point-of-care testing led by pharmacists, using a PICCOLO device to measure eGFR, successfully identified a large proportion of undiagnosed CKD cases (13). Comparable approaches have been trialled in India and Thailand, where validated point-of-care strategies for microalbuminuria and renal risk factors enhanced early CKD detection in both rural and urban populations (31, 32). Implementing such strategies in Arab countries, particularly those with constrained resources, may improve early detection and facilitate timely interventions.

Hypertension has consistently been established as a primary driver of CKD progression. The included studies reaffirm this association, identifying HTN as both a precursor to and a consequence of CKD (13, 14, 19, 20). Additional risk factors include diabetes, obesity, cardiovascular disease, smoking, male sex, unemployment, and dyslipidaemia (16, 33, 34). In Iraq, Kamil et al. (2021) found that over 75% of CKD patients had a history of HTN, with men significantly more affected than women (17). Although CKD prevalence and incidence are often higher in women, men experience more rapid disease progression and worse clinical outcomes, due to factors such as delayed healthcare-seeking, lower treatment adherence, and hormonal influences (27, 28, 35).

Suboptimal blood pressure control also contributes to CKD progression. In Iraq, Nassr and Forsyth (2019) reported that fewer than 40% of hypertensive patients achieved controlled BP, indicating poor adherence to clinical guidelines (22). Pharmacological management practices remain variable across the region. While the use of renin-angiotensin-aldosterone system (RAAS) inhibitors is recommended, their prescription remains inconsistent. Al-Shamsi et al. (2016) reported that only 76% of physicians prescribed ACE inhibitors or ARBs, and many estimated that less than half of their patients were adherent to these therapies (18). These findings point to persistent gaps between knowledge and practice in the management of HTN and CKD.

Socioeconomic and structural factors further exacerbate the burden of CKD in the region. Despite the presence of several high-income countries, the Arab world faces significant challenges in delivering equitable kidney care. These include health system fragility, resource constraints, limited public awareness, ongoing conflict, and environmental stressors such as heat, water scarcity, air pollution, and disparities in healthcare access (36, 37). Cultural norms and low health literacy also hinder screening and treatment adherence, particularly in marginalised communities.

Addressing these challenges requires comprehensive, context-specific strategies. Alsheikh et al., 2025 have called for public health campaigns promoting early screening, lifestyle interventions, and education tailored to local contexts (38). Integrating person-centred care into primary health systems, as advocated by Rawaf et al. (2023), could significantly enhance engagement, adherence, and outcomes for patients with HTN and CKD (39). Actionable priorities for Arab-country health systems include: (i) embedding combined eGFR and albuminuria testing within primary care and national NCD screening pathways for hypertensive adults (9, 30); (ii) strengthening non-nephrologist training and clear referral pathways for CKD staging and risk stratification (18, 30); (iii) improving BP control through guideline-concordant prescribing and adherence support, including appropriate RAAS inhibitor use where indicated (6, 18); and (iv) monitoring HTN-related CKD indicators within national NCD surveillance to track detection, treatment, and progression (9, 10, 12). Community-based screening models, such as pharmacist-led point-of-care approaches, may complement formal programmes in settings where access and follow-up are constrained (13, 36, 37).

Despite its contributions, this review has limitations. The included studies were heterogeneous in methodology, scope, and reporting quality, which prevented us from conducting a meta-analysis with the extracted results. The included studies differed substantially in population characteristics, sampling frames (community, primary care, outpatient and hospital-based settings), outcome definitions (eGFR-only versus combined eGFR and albuminuria/proteinuria), and reporting of effect measures. In addition, several studies did not provide sufficiently comparable numerators/denominators or stratified outcomes (e.g., by CKD stage or HTN status) to support pooled estimates or risk ratio calculations. Several relied on older data or lacked detailed stratification by CKD stage or treatment outcomes. Furthermore, the review revealed a notable gap in well-designed intervention studies or evaluations of national CKD prevention programmes in Arab countries. Future research should prioritise longitudinal designs, standardised diagnostic criteria, and policy-relevant interventions to control HTN and slow CKD progression.

## Conclusion

This review highlights the substantial and growing burden of CKD among HTN populations in Arab countries. HTN, along with metabolic and sociodemographic risk factors, significantly contributes to disease development and progression. Early detection remains hindered by diagnostic limitations, provider practices, and systemic challenges. Multisectoral efforts, encompassing clinical, community, and policy-based strategies, are urgently needed to enhance CKD prevention through screening, optimise HTN management, and improve population health outcomes in the region.

## Data Availability

The original contributions presented in the study are included in the article/**Supplementary Material**. Further inquiries can be directed to the corresponding author.
