# Supplementary material for: Chronic kidney disease in hypertensive patients: the urgent need for targeted interventions in Arab countries: a systematic review
Source: Front Nephrol. 2026 Feb 9;6:1735217. doi: 10.3389/fneph.2026.1735217 (PMC12926114; doi:10.3389/fneph.2026.1735217)
Supplement: Supplementary file 1 [file DataSheet1.docx]

**Database Search Terms:**

- **Scopus:**

( TITLE-ABS-KEY ( "chronic kidney disease" OR "renal disease" ) AND TITLE-ABS-KEY ( "hypertension" OR "essential hypertension" OR "systolic hypertension" ) AND TITLE-ABS-KEY ( "prevent*" OR "manag*" ) AND TITLE-ABS-KEY ( "primary care" ) AND TITLE-ABS-KEY ( "Comoros" OR "Djibouti" OR "Mauritania" OR "Yemen" OR "Somalia" OR "Algeria" OR "Egypt" OR "Iraq" OR "Jordan" OR "Lebanon" OR "Libya" OR "Morocco" OR "Palestin*" OR "Sudan" OR "Syria" OR "Tunisia" OR "Bahrain" OR "Kuwait" OR "Oman" OR "Qatar" OR "Saudi Arabia" OR "United arab emirates" OR "UAE" OR "KSA" ) )

**- Medline/ Embase (Ovid Software):**

1. (chronic kidney disease or Renal disease).af.

2. (hypertension or essential hypertension or systolic hypertension or blood pressure).af.

3. 1 and 2

4. (prevent* or manag* or target or control).af.

5. 3 and 4

6. primary care.af.

7. 5 and 6

8. (Comoros or Djibouti or Mauritania or Yemen or Somalia or Algeria or Egypt or Iraq or Jordan or Lebanon or Libya or Morocco or Palestin* or Sudan or Syria or Tunisia or Bahrain or Kuwait or Oman or Qatar or Saudi Arabia or United arab emirates or UAE or KSA).

9. 7 and 8

- **PubMed:**

( "chronic kidney disease" OR "renal disease" ) AND TITLE-ABS-KEY ( "hypertension" OR "essential hypertension" OR "systolic hypertension" ) AND TITLE-ABS-KEY ( "prevent*" OR "manag*" ) AND TITLE-ABS-KEY ( "primary care" ) AND TITLE-ABS-KEY ( "Comoros" OR "Djibouti" OR "Mauritania" OR "Yemen" OR "Somalia" OR "Algeria" OR "Egypt" OR "Iraq" OR "Jordan" OR "Lebanon" OR "Libya" OR "Morocco" OR "Palestin*" OR "Sudan" OR "Syria" OR "Tunisia" OR "Bahrain" OR "Kuwait" OR "Oman" OR "Qatar" OR "Saudi Arabia" OR "United arab emirates" OR "UAE" OR "KSA" )

- **Cochrane Library Search:**

("chronic kidney disease" OR "renal disease"):ti,ab,kw AND

("hypertension" OR "essential hypertension" OR "systolic hypertension"):ti,ab,kw AND

(prevent* OR manag* OR target OR control):ti,ab,kw AND

("primary care"):ti,ab,kw AND

("Comoros" OR "Djibouti" OR "Mauritania" OR "Yemen" OR "Somalia" OR "Algeria" OR "Egypt" OR "Iraq" OR "Jordan" OR "Lebanon" OR "Libya" OR "Morocco" OR "Palestin*" OR "Sudan" OR "Syria" OR "Tunisia" OR "Bahrain" OR "Kuwait" OR "Oman" OR "Qatar" OR "Saudi Arabia" OR "United Arab Emirates" OR "UAE" OR "KSA"):ti,ab,kw
