# Supplementary material for: Chronic kidney disease in hypertensive patients: the urgent need for targeted interventions in Arab countries: a systematic review
Source: Front Nephrol. 2026 Feb 9;6:1735217. doi: 10.3389/fneph.2026.1735217 (PMC12926114; doi:10.3389/fneph.2026.1735217)
Supplement: Supplementary file 2 [file DataSheet2.docx]

Supplementary 2

Quality assessment template for cross-sectional study based on Newcastle-Ottawa-Scale (NOS)

| Author | Year |  |  | Selection Bias Assessment | | |  |  |  | Comparability | | Outcome (Maximum 3 stars) | | | |  |
| --- | --- | --- | --- | --- | --- | --- | --- | --- | --- | --- | --- | --- | --- | --- | --- | --- |
|  |  |  |  |  |  |  |  |  |  | (Maximum 2 stars) | |  |  |  |  |  |
|  |  |  |  | (Maximum 5 stars) | | |  |  |  |  |  |  |  |  |  |  |
|  |  |  |  |  |  |  |  |  |  |  | |  |  |  |  |  |
|  |  | Representativeness | | Sample size | | Non-respondents | | Ascertainment of | | Confounding | | Assessment of | | Statistical Test | | Total score |
|  |  | of the sample | |  |  |  |  | the exposure (risk | | factors are | | the outcome | |  |  | (Maximum |
|  |  |  |  |  |  |  |  | factor) | | controlled | |  |  |  |  | 10 stars) |
|  |  | selection | score | selection | score | selection | score | selection | score | selection | score | selection | score | selection | score |  |
| Ahmed Abdulrahman Aldhahi | 2018 | Yes/1 | | Yes/2 | | No/1 | | Yes/1 | | Yes/1 | | Yes/1 | | Yes/2 | | 9 |
| [Hussain Gadelkarim Ahmed1](https://eajbsc.journals.ekb.eg/article_16028.html) | 2013 | Yes/2 | | Yes/2 | | Yes/1 | | Yes/1 | | Yes/1 | | Yes/1 | | Yes/1 | | 9 |
| [Hala H. Sa’adeh1](https://pmc.ncbi.nlm.nih.gov/articles/PMC5885300/) | 2017 | Yes/2 | | Yes/2 | | Yes/1 | | Yes/1 | | Yes/2 | | Yes/1 | | Yes/2 | | 10 |
| Amani A. Khalil | 2014 | Yes/2 | | Yes/2 | | Yes/1 | | Yes/1 | | Yes/1 | | Yes/1 | | Yes/1 | | 9 |
| [Ali Manal Kamil1](https://www.researchgate.net/publication/350088913_Prevalence_of_chronic_kidney_disease_and_hypertension_as_a_risk_factor_in_Basrah_province_-_Iraq) | 2019 | Yes/1 | | Yes/2 | | Yes/1 | | No/0 | | Yes/1 | | Yes/1 | | Yes/1 | | 7 |
| Nassr | 2019 | Yes/2 | | Yes/1 | | No/1 | | Yes/1 | | Yes/2 | | Yes/1 | | Yes/2 | | 10 |
| AlShamsi | 2016 | Yes/2 | | Yes/1 | | Yes/1 | | Yes/1 | | Yes/1 | | Yes/1 | | Yes/2 | | 9 |
| Jairoun | 2024 | Yes/1 | | Yes/1 | | No/2 | | Yes/1 | | Yes/1 | | Yes/1 | | Yes/2 | | 9 |

Quality assessment template for cohort study based on Newcastle-Ottawa Scale (NOS)

| Author | Year |  |  | Selection Bias Assessment | | |  |  |  | Comparability | | Outcome (Maximum 3 stars) | | | | | |  |
| --- | --- | --- | --- | --- | --- | --- | --- | --- | --- | --- | --- | --- | --- | --- | --- | --- | --- | --- |
|  |  |  |  |  |  |  |  |  |  | (Maximum 2 | |  |  |  |  |  |  |  |
|  |  |  |  | (Maximum 4 stars) | | |  |  |  |  |  |  |  |  |  |  |  |  |
|  |  |  |  |  |  |  |  |  |  | stars) | |  |  |  |  |  |  |  |
|  |  |  | |  | |  | | Demonstration | | Comparability of | |  | | Was follow-up | |  | | Total |
|  |  | Representativeness | | Selection of the | |  | | that outcome of | |  |  |  | |  |  | Adequacy of | |  |
|  |  |  |  |  |  | Ascertainment of | |  |  | cohorts on the | | Assessment of | | long enough for | |  |  | score |
|  |  | of the exposed | | non-exposed | | exposure | | interest was not | | basis of the | | the outcome | | outcomes to | | follow up of | | (Maximum |
|  |  | cohort |  | cohort | |  |  | present at start | |  |  |  |  |  |  | cohorts | |  |
|  |  |  |  |  |  |  | |  |  | design or analysis | |  | | occur | |  |  | 10 stars) |
|  |  |  |  |  | |  | | of study | |  |  |  | |  |  |  | |  |
|  |  | selection | score | selection | score | selection | score | selection | score | selection | score | selection | score | selection | score | selection | score |  |
| [S Al-Shamsi](https://pubmed.ncbi.nlm.nih.gov/29949629/) | 2018 | Yes/2 | | No/0 | | Yes/1 | | Yes/1 | | Yes/1 | | Yes/1 | | Yes/1 | | Yes/1 | | 8 |

| Author | Year |  |  | Selection Bias Assessment | | |  |  |  | Comparability | | Outcome (Maximum 3 stars) | | | | | |  |
| --- | --- | --- | --- | --- | --- | --- | --- | --- | --- | --- | --- | --- | --- | --- | --- | --- | --- | --- |
|  |  |  |  |  |  |  |  |  |  | (Maximum 2 stars) | |  |  |  |  |  |  |  |
|  |  |  |  | (Maximum 4 stars) | | |  |  |  |  |  |  |  |  |  |  |  |  |
|  |  |  | |  | |  | |  | | Comparability of | |  | | Same method of ascertainment for cases and controls | |  | | Total |
|  |  | Is the case definition adequate? | |  | |  | |  | | cases and controls on the basis of the design or analysis | |  | |  |  |  | |  |
|  |  |  |  | Representativeness  Of the cases | | Selection of controls | | Definition of controls | |  |  | Assessment of the exposure | | ascertainment | | Non-response rate | | score (Max 10 stars) |
|  |  |  |  |  | |  | |  | |  |  |  | |  | |  |  | 10 |
|  |  | selection | score | selection | score | selection | score | selection | score | selection | score | selection | score | selection | score | selection | score |  |
| Hassanien | 2014 | Yes/1 | | Yes/2 | | Yes/1 | | Yes/1 | | Yes/1 | | Yes/1 | | Yes/1 | | No/1 | | 9 |
| Nasser | 2010 | Yes/1 | | Yes/1 | | Yes/1 | | Yes/1 | | Yes/1 | | Yes/1 | | Yes/1 | | No/1 | | 8 |
